# A Robotic Flexible Drill and Its Navigation System for Total Hip Arthroplasty

**DOI:** 10.1007/s10439-017-1959-5

**Published:** 2017-11-22

**Authors:** Ahmad Nazmi Bin Ahmad Fuad, Hariprashanth Elangovan, Kamal Deep, Wei Yao

**Affiliations:** 1 The Department of Biomedical Engineering, University of Strathclyde, 106 Rottenrow, Glasgow, G4 0NW Scotland, UK; 2 Golden Jubilee National Hospital, Agamemnon Street, Clydebank, G81 4DY Scotland, UK

**Keywords:** Robotics, Flexible, Steerable, Tracking, Navigation, Total hip arthroplasty (THA), Orthopaedics

## Abstract

This paper presents a robotic flexible drill and its navigation system for total hip arthroplasty (THA). The new robotic system provides an unprecedented and unique capability to perform curved femoral milling under the guidance of a multimodality navigation system. The robotic system consists of three components. Firstly, a flexible drill manipulator comprises multiple rigid segments that act as a sheath to a flexible shaft with a drill/burr attached to the end. The second part of the robotic system is a hybrid tracking system that consists of an optical tracking system and a position tracking system. Optical tracking units are used to track the surgical objects and tools outside the drilling area, while a rotary encoder placed at each joint of the sheath is synchronized to provide the position information for the flexible manipulator with its virtual object. Finally, the flexible drill is integrated into a computer-aided navigation system. The navigation system provides real time guidance to a surgeon during the procedure. The flexible drill system is then able to implement THA by bone milling. The final section of this paper is an evaluation of the flexible and steerable drill and its navigation system for femoral bone milling in sawbones.

**Electronic supplementary material:**

The online version of this article (10.1007/s10439-017-1959-5) contains supplementary material, which is available to authorized users.

## Introduction

Surgical robotic technology has been developing for decades to the extent that many surgical practices now benefit from the deployment of surgical robotic platforms. These benefits include increasing the accuracy, minimizing complications of surgery and improving patient outcomes. In orthopaedic surgery, computer-aided orthopaedic surgery (CAOS) has been advancing by using robotic surgical devices and navigation systems, resulting in a great improvement of surgical field visibility and the enhancement of surgical accuracy.^21^ In particular, the use of robotic surgical devices for orthopaedic surgery has become more widely accepted for its greater precision and accuracy in implant positioning and orientation.^16^ There are three types of surgical robotic systems that have been developed for orthopaedics surgery, which are passive, semi-active and active systems. Passive systems control surgical tools by moving a cutting guide block or a drilling guide sleeve while a surgeon handles the tool with his free hands.^12^,^28^ Semi-active systems limit the movement of surgical tools within a pre-operative planned surgical area by means of a robot arm, examples include MAKO and ACROBOT.^11^,^29^ Active systems such as ROBODOC and CASPAR can execute surgical planning automatically, independent of the surgeon’s hands.^4^,^26^

In robotic orthopaedic surgery, the surgeon needs to perform procedures precisely and safely within a limited space; and this requires effective surgical guidance by means of a surgical navigation. Surgical navigation in orthopaedic surgery works on a similar principle using a global positioning satellite (GPS), in which a virtual visualization of the surgical tools and the anatomy is operated on in real-time, acting as a guide for the surgeon during surgical procedures. Advancement in radiographic imaging enables the reconstruction of imaging data into three dimensional (3D) images, which can be used in surgical pre-operative planning to various surgical procedures.^6^ The digital radiographic images serve as a navigation map for the procedures, where the surgical tools’ CAD models are incorporated into the map for the purpose of visualizing its position, orientation and its movement to an accuracy of one millimetre or one degree.^27^ This method greatly improves the accuracy and precision of the surgery and gives surgeon a better and wider view of surgical field.

The advent of computer-aided surgery (CAS) and surgical robotics has given a lot of improvement to minimal invasive surgery (MIS). It enhances three advantages of MIS over conventional surgery, which are free manoeuvrability for the instrument, sensory feedback and three-dimensional imaging.^23^ However, the advantages of free manoeuvrability for the instrument are still not fully developed since some areas in surgical operations are still not accessible *via* rigid surgical tools, or the current surgical tools are not accurate enough for MIS.^8^ Thus, flexible surgical tools have been investigated. Through the use of these flexible tools, surgeons can access problematic zones, such as sinuses in endonasal sinus surgery, visualise hidden tissue structures in arthroscopy, and a curved-drilling approach in core decompression of the femoral head osteonecrosis.^1^ Some flexible tools have also been demonstrated to be able to control a needle puncture and penetrate tissues from any point within the body such as in tissue biopsies^22^; reduce insertion forces and prevent buckling by using robot-assisted and steerable electrode prototypes in cochlear implant surgery^31^; and carry out vascular catheterization to treat cardiac and vasculature disease.^5^ However, there are not any flexible tools available that are able to provide sufficient precision and force transformation for bone milling in orthopaedic surgery.

Volumetric-based navigation has been used in total hip arthroplasty (THA),^9^ total knee arthroplasty,^3^,^10^,^17^,^24^ pelvic osteotomy and in spine screw insertion in spinal surgery.^2^,^13^ These methods give surgeons better visualization of complete constructs/attachments between implant and bone. The total joint arthroplasty procedures utilize both 3D CAD and volume rendering bone models allowing surgeons to pre-operatively simulate a range of motions of the joints.^29^ There are three major components in surgical navigation systems. Surgical object (SO) means the anatomical location of surgical action. Virtual object (VO) includes a virtual representation of a surgical object that allows surgeon to plan the surgical procedure before the actual surgery and execute its intra-operatively. Navigator (NAV) is a device that establishes the coordinate systems (COS) of the surgical field targets and the location and orientation of utilized end-effectors (EE).^21^ In order to fully utilize the surgical navigation system, certain processes are required to setup the system. Those are calibration of end-effectors, registration, and dynamic referencing. Calibration for end-effector is required to describe its geometry in the coordinate of the navigator. To calibrate the end-effector, a rigid attachment of optical markers is introduced in the optical tracking system. Registration is a process that a surgical navigation system links the SO and VO in real time allowing them to display on the monitor. It is realized by surgeons’ identifying key anatomical landmarks resulting in better accuracy of the alignment. Dynamic referencing is one of important requirement for a surgical navigation system. It is necessary for positioning control compensation of a possible motion of the navigator and/or surgical objects during the surgical procedures. This is established by attaching dynamic referencing bases (DRBs), which consist of three more reflective markers or light emitting diodes (LEDs) arranged in a pattern at the surgical device so that it acts as the base of reference to other surgical objects in tracking. The DRBs can either be fixed to the patient representing fixed anatomy or it can be mobile when attached to surgical tools.

In THA, robotic orthopaedic surgery is only practiced with acetabular cup positioning and orientation; however, femoral stem positioning still uses the hand-rasping method instead of femoral milling, because current rigid tools are not able to drill through curved pathways. To reduce trauma, minimally invasive procedures are increasingly demanded for THA surgery. There are advantages in using femoral milling in a minimally invasive procedure compared to using the hand-rasping method, such as the prevention of intra-operative fractures and providing better fit with less trauma.^20^ However, femoral milling is not widely practiced due to the space-constraints in MIS. Although some studies have been reported on the utilization of robotic surgical systems for both acetabular cup and femoral stem implantation, this is only for implementing the normal open approach rather than the MIS approach.^25^ This minimally invasive procedure needs a more dexterous manipulator for femoral milling. The emergence of robotic technology gives us an opportunity for developing a flexible and steerable drill tip, which can be integrated into a computer-aided surgical system. The following Table 1 shows all other robotic orthopaedic systems are using rigid drill and not tracked and navigated inside the bone.

**Table 1:** Current robotics orthopaedic systems.

Robotic/steerable drill	Manipulator	Tracking	Navigation	Control
BlueBlet^28^	Rigid	Optical tracking	Virtual model	Free-hand
MAKO^29^	Rigid with haptics	Optical tracking	Virtual model	Semi-active
ACROBOT^11^	Rigid	Mechanical tracking	Over-constrain	Semi-active
ROBODOC^4^	Rigid	Mechanical tracking	No	Active
CASPAR^26^	Rigid	Mechanical tracking	No	Active
Continuum Manipulator^1^	Flexible	X-ray	X-ray	Passive
Our flexible drill	Flexible	Optical tracking + kinematic tracking	Virtual model	Free-hand

This paper presents a novel flexible robotic system that includes a flexible drill manipulator, a hybrid tracking device and a multi-modality navigation system. The system enables, for the first time, to have the capability of guiding the curved 3D milling inside the bone. This paper is organized as follows: in the first section, the need for a flexible surgical drill for orthopedic surgery is presented and a concept design is introduced; next section follows a detailed flexible drill design and its tracking and navigation system; then, a test rig is established to evaluate the system in sawbones.

## Materials and Methods

### Concept Design of the Flexible Robotic System

**Figure 1:**
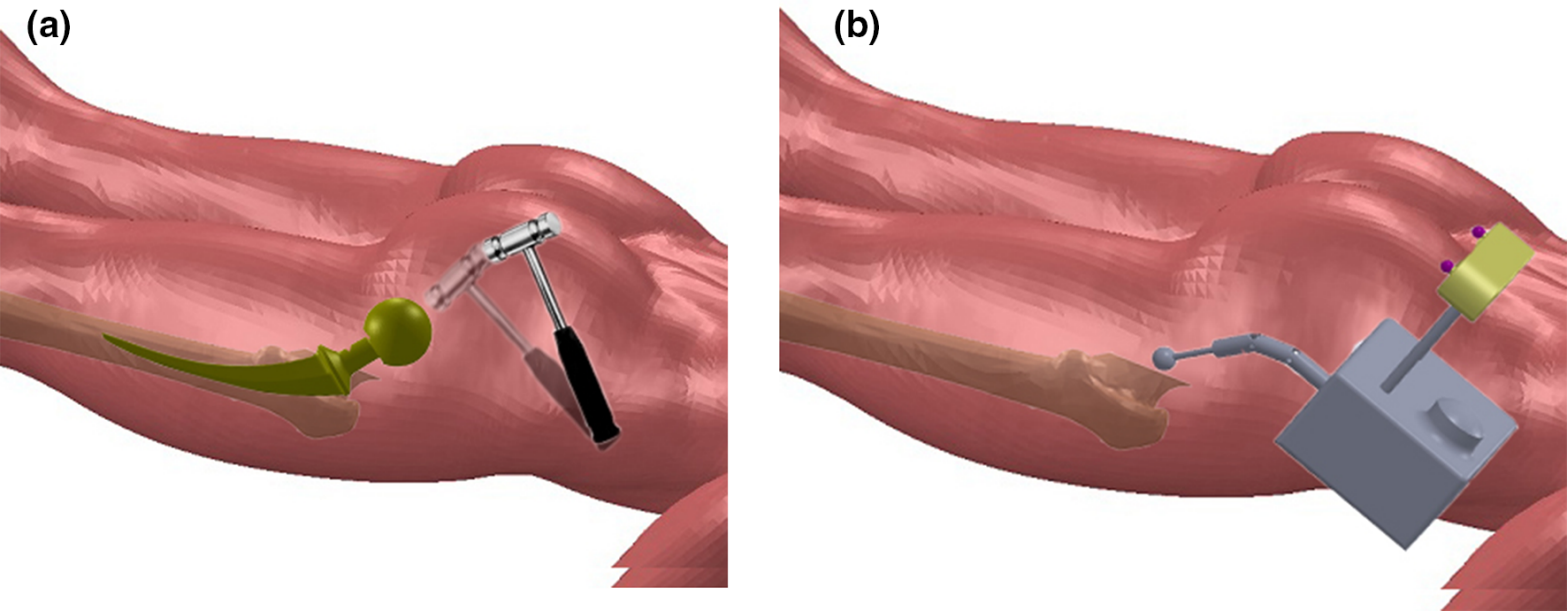
(**a**) Traditional hand-rasping method for Total Hip Arthroplasty; (**b**) proposed method by using the robotic flexible drill.

The research is driven by the clinical requirements in improving the accuracy and reducing the trauma in orthopedic surgery. Firstly, shown in Fig. 1, conventional surgery for joint replacement is currently not very accurate as its hand-rasping method. In the case of THA, since femoral stem is slightly curved, in order to follow the anatomical shape of femur, a new flexible surgical drill is required to mill a curved femoral canal under the guidance of its navigation system for THA. In addition, the flexible drill can benefit patients by adopting a minimal invasive approach due to its variable bending configuration.

**Figure 2:**
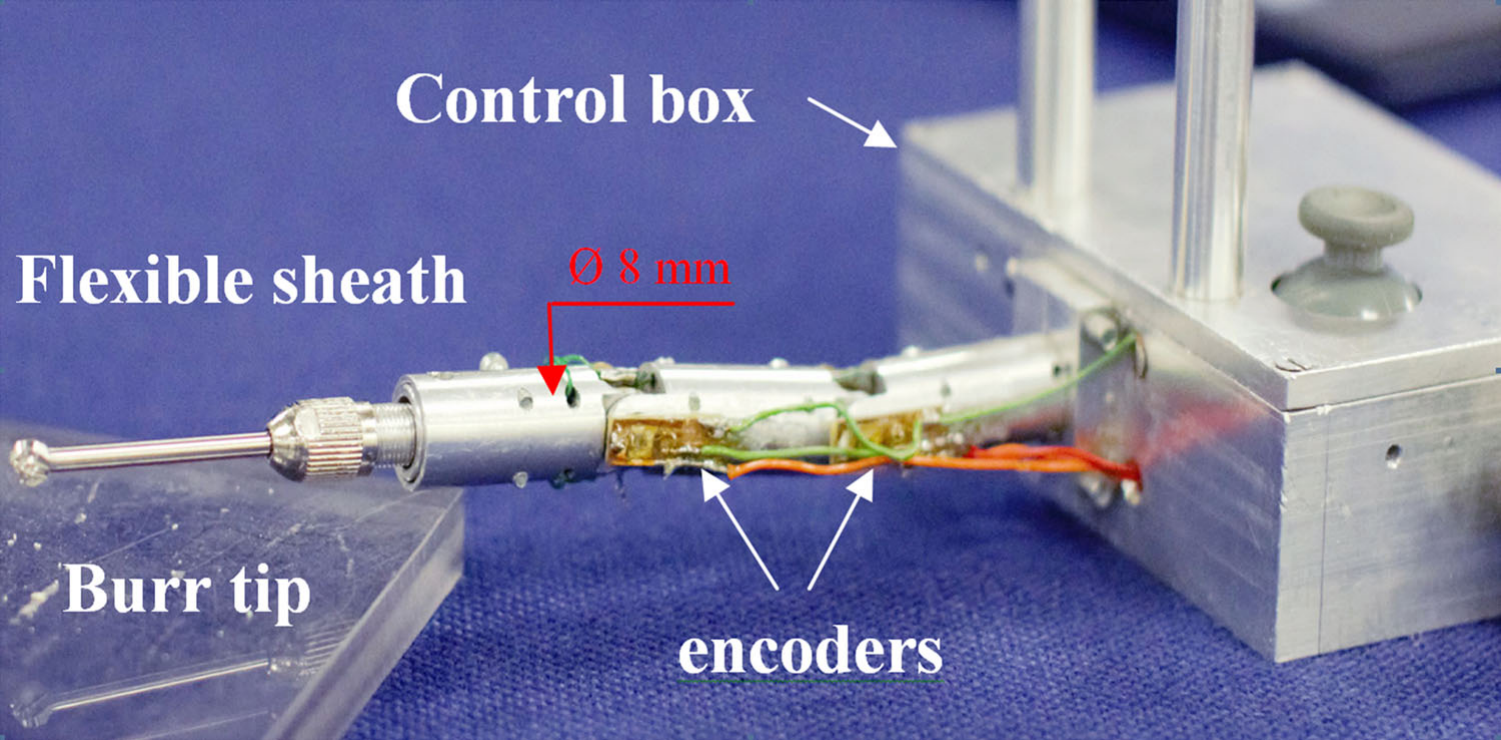
A prototype of the flexible drill mechanism.

The concept design of the system shown in Fig. 2 consists of a novel flexible drill to enable intra-operative tunnelling and a navigation system for tracking and navigating for the end-effector (EE) inside the bone. Due to the fact that the flexible drill tip is not trackable *via* current optical tracking systems, this research focuses on developing a hybrid tracking system for the flexible drill by integrating optical tracking devices and position sensors in the flexible tips. The optical tracking system tracks the surgical tools outside the drilling canal, while rotary encoders are used to track the end of the flexible drill tip. A navigation system of the new robotic system guides the procedure by providing a real-time virtual model of the flexible drill and its association with a CAD bone model from a CT scan. The flexible drill system is then experimented in sawbones, followed by an evaluation of the positioning of femoral stem placement by femoral milling. This system demonstrates an innovative robotic platform designed to allow surgeons to achieve a new level of precision and flexibility.

The manipulator is required to fit within a small incision inside the femoral canal, to be able to bend for the milling of the curved shape in the femur, and to be rigid enough to make femoral canal possible without any buckling at the base.

### Design of a Novel Flexible Drill Mechanism

The flexible drill comprises three multiple rigid segments that act as a sheath to a flexible shaft with a drill/burr attached to the end, as illustrated in Fig. 2. The outer diameter of the sheath is 8 mm; the length of the sheath to the burr tip is 158.5 mm. The proximal end segment is connected to the motor box as shown in Fig. 2, in which the actuation of the flexible drill takes place. The motor box is designed to be a handle with a servo motor and a microcontroller board fitted in. The microcontroller controls the servo and streams data from the rotary encoders. The revolution joint connects each two segments linked by two rivets that allow free rotation of the joint. Inside the sheath, two ball bearings are installed to link each half of the sheath to the flexible shaft that drives the drilling with the maximal speed of 30,000 rpm. This design allows the drill mechanism to have a free rotation and strong force transmission. The 3 mm flexible shaft runs through a 5 mm hole at the base. The flexible drill has a wire-driven steering capability for bending the joints. Two channels are designed for wires that connect the drill end part to the servo motor with the torque value of 11.3 kg/cm at 6.0 V, enabling bending of the joints in clockwise and counter-clockwise directions.

The bending is controlled by wires pulling inside the drill sheath. This design enables the drill to navigate through the small incision inside the femoral canal.

**Figure 3:**
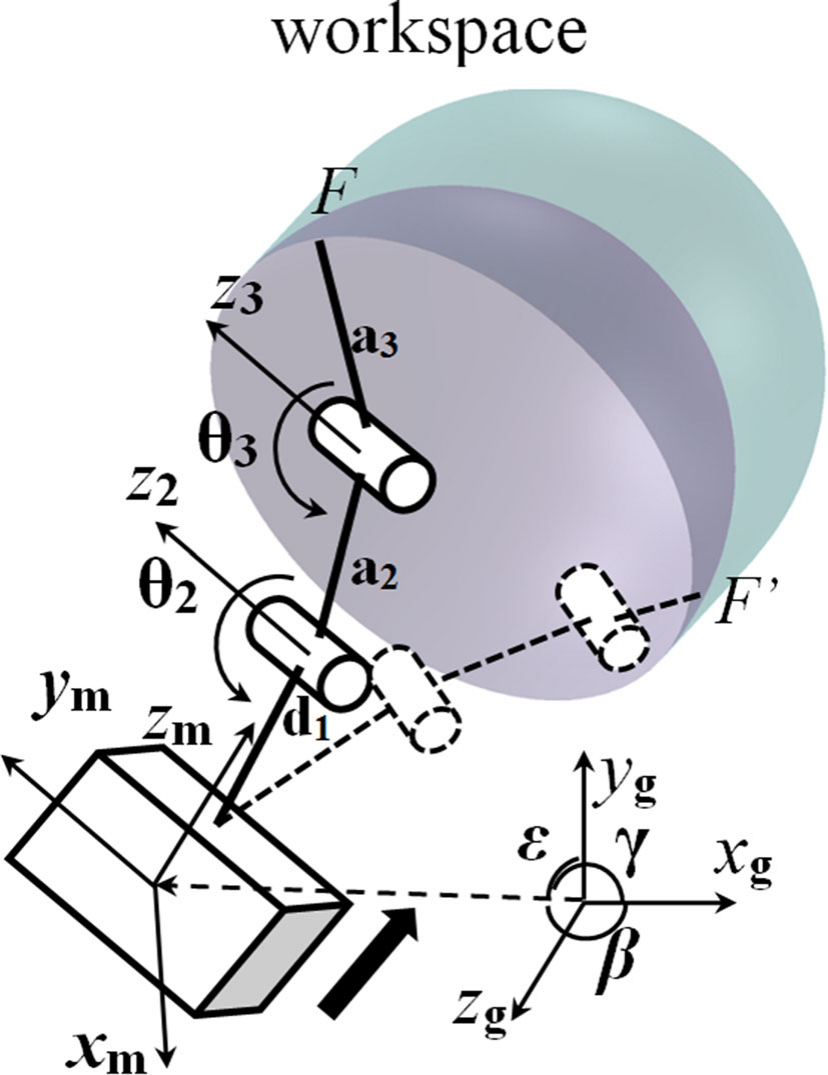
Kinematic sketch and the workspace of the flexible drill.

A three-bar kinematic chain is designed as the flexible drill mechanism from the kinematic sketch of the mechanism in Fig. 3. The drill mechanism is designed as a kinematic chain with three binary links attached by two revolute joints, allowing one link to rotate with respect to the other links. The kinematics of the manipulator is calculated using D–H (Denawit–Hartenberg) parameters. In the kinematics of the flexible drill manipulator, each *T*_*i*_ is defined by two parameters, *a*_*i* − 1_ and *θ*_*i*_. It is expressed by moving *A*_*i*_ from its own body frame onto the body frame of *A*_*i* − 1_. Furthermore, the combinational transformation matrix *T*_*i* − 1_ *T*_*i*_, can be approached by moving both *A*_*i*_ and *A*_*i* − 1_ to the body frame *A*_*i* − 2_. The resulting equation is as below,

1 $$T_{i} = \left( {\begin{array}{cccc} {\cos \theta_{i} }&\quad {- { \sin }\theta_{i} } &\quad 0 &\quad {\alpha_{i - 1} } \\ {{ \sin }\theta_{i} } &\quad{\cos \theta_{i} } &\quad0 &\quad0 \\ 0 &\quad0 &\quad1 &\quad{d_{i} } \\ 0 &\quad0 &\quad0 &\quad1 \\ \end{array} } \right)$$

In the kinematic analysis, *T*_*m*_ will be defined as a rigid-body homogenous transformation matrix and this represents the six degrees of freedom of the free handle that is tracked by the optical tracking device. The rigid-body homogeneous transformation matrix is a 4 × 4 matrix that performs the rotation given by *R* (*β,γ,ε*), followed by a translation given by *x*_*m*_, *y*_*m*_, *z*_*m*_. This results in the homogeneous transformation matrix *T*_*m*_,

2 $$T_{m} = \left( {\begin{array}{*{20}c} {\cos \beta \cos \gamma } & {\cos \beta \sin \gamma \sin \varepsilon - \sin \beta \cos \varepsilon } & {\cos \beta \sin \gamma \cos \varepsilon + \sin \beta \sin \varepsilon } & {x_{m} } \\ {\sin \beta \cos \gamma } & {\sin \beta \sin \gamma \sin \varepsilon + \cos \beta \cos \varepsilon } & {\sin \beta \sin \gamma \cos \varepsilon - \cos \beta \sin \varepsilon } & {y_{m} } \\ { - \sin \gamma } & {\cos \gamma \sin \varepsilon } & {\cos \gamma \cos \varepsilon } & {z_{m} } \\ 0 & 0 & 0 & 1 \\ \end{array} } \right)$$

The end-effecter *F* in the body frame of the last link *A*_3_ appears in the coordination of G as

3 $$T_{F} = T_{m} T_{1} T_{2} T_{3}$$

where, *G* denotes the global coordinate system of the navigation system.

Although the flexible sheath is three links, it provides one more DOF as it is actuated by one tendon mechanism for bending the flexible shaft with a drill/burr tip attached. The maximal bending angle is limited by 90° between the tip and the base of the flexible sheath. As the maximal bending angle is 90° the workspace is a half circle when the handle is fixed. The 3-link PRR manipulator is based on a 6 DOFs “freehand” handle; in minimally invasive Total Hip Arthroplasty, as the entry space is very limited, handle motion might only be allowed to push along and rotate the axis of the fix part of the flexible shaft. In addition with the extra bending of the flexible sheath, the workspace would be a double half sphere described in the Fig. 3. This workspace also shows the flexibility of the manipulator. Thus, it can tunnel a curved canal inside the femur which makes the implantation more precise.

### A Multi-modality Tracking System

**Figure 4:**
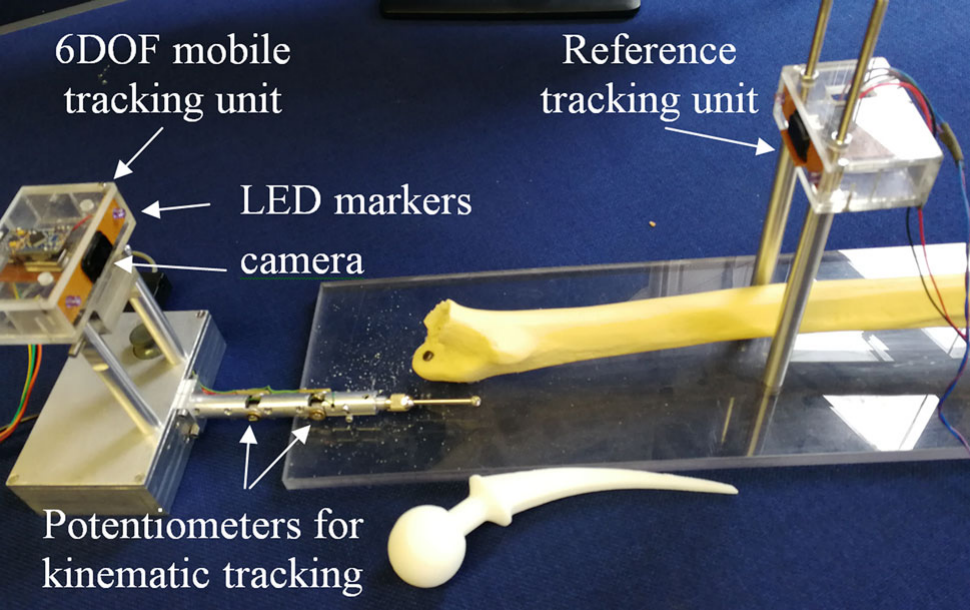
The setting of the multi-modality tracking system.

The second part of the robotic drill system is a multi-modality tracking system for the flexible drill that integrates an optical tracking system and a rotary encoder-based tracking system. An optical tracking unit is mounted at the base of the manipulator to track its position and orientation, while the potentiometer placed at each joint of the sheath provides bending angle for tracking the end of the flexible drill tip inside the drilling canal.^30^ As the optical tracking system can only track the open part of the flexible drill mechanism, when the tip of the flexible drill tunnels inside the bone, the potentiometer-based tracking system is combined to provide the completed position information for the flexible drill. The optical tracking system consists of beacons of two infrared LED trackers and a small infrared camera at the middle of the LED trackers. The beacons contain infrared LEDs arranged in a specific pattern and act as a stationary reference plane. Micro infrared cameras interpolate to give 1024 × 768 pixels, at a frame rate of up to 100 frames per second. They act as mobile/independent trackers, and are attached to each surgical object and the end-effector (Fig. 4). The following Figure shows how the different types of tracking units are set up in the multi-modality tracking system.

The flexible drill sheath to be tracked inside the bone is set with encoders attached at each joint of the sheath. The potentiometers function as rotary encoders that measure the bending angle of each of the joints. They determine the position of the burr tip with reference to the base of the drill manipulator calculated by its forward kinematics. The bending angle of each of the flexible drill sheath joints is equal to the rotational angle of the encoder’s shaft. Hence, the voltage output of the encoder at each degree of rotation is taken and mapped as a bending angle of the joints. Analog data from the encoders is connected to a microcontroller board that converts it to digital data. The data is then read by the navigation system as rotation angle for joints. The angle data, combined with the length of each segment is then used to map the position of the flexible sheath location and to synchronize it with its virtual object. This tracking system tracks and updates the virtual object to guide the surgical procedure.

### Navigation System

The flexible drill is integrated into a computer-aided navigation system. First, a mapping system is developed by acquiring 3D images of a femur and a femoral stem implant. A femur 3D model is obtained by scanning a femur in a CT scanner. This model is then imported to create a 3D mesh model. The mapping enables a surgeon to virtually view both 3D model of a bone and 3D model of a femoral stem, thus enabling surgeon to plan the position of femoral stem inside bone model. Also, the coordinates of both the femur and femoral stem models can be linked together to enable virtual interaction between the models.

**Figure 5:**
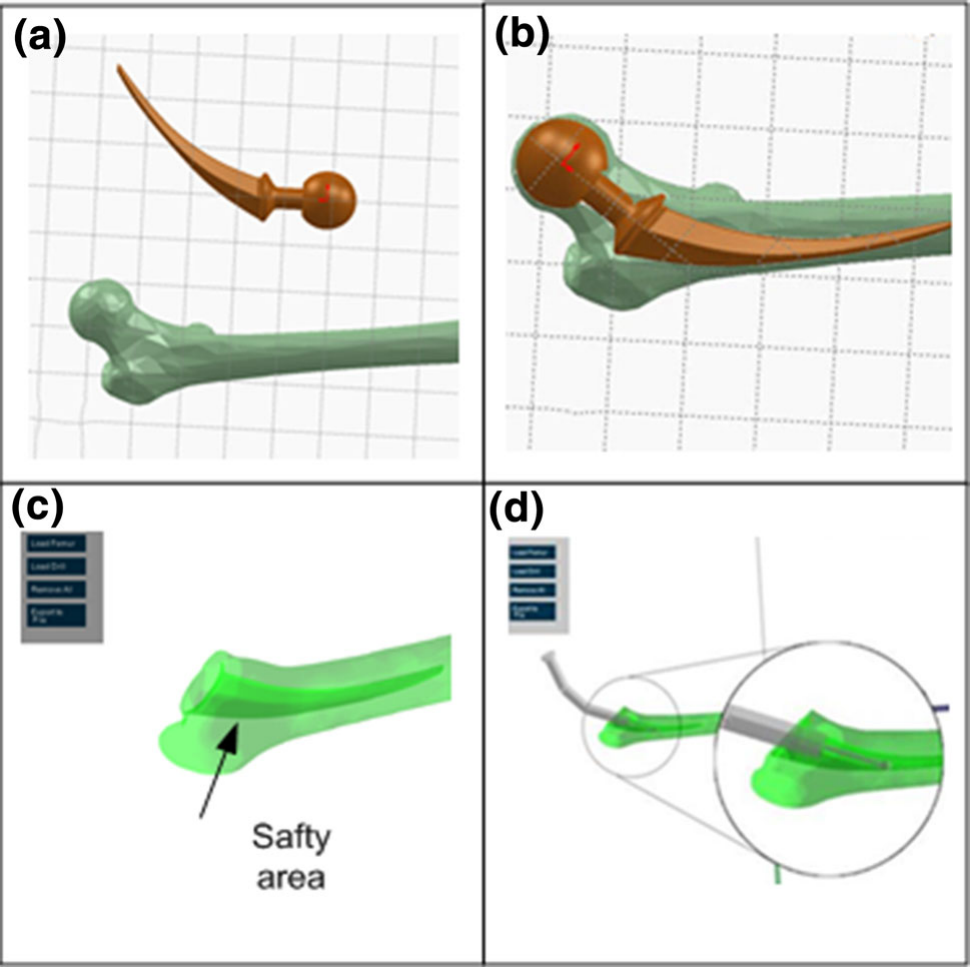
Setting up the boundary of the safe surgical volume (**a**) The femur and implant 3D models; (**b**) Position the femoral stem inside the 3D bone model; (**c**) Safety area is defined as the deeper green area; (**d**) The milling is guided by the safety boundary.

The next step is to set up the boundary of safe surgical volume in Fig. 5. The safe surgical volume is milled to confine the volume of femoral stem model. Thus, the 3D femoral stem model is transferred to be a boundary of the safe surgical volume. The burring motor is designed to stop once the burr tip reaches the boundary. Setting up the boundaries enables precise milling that follows the shape of the implants. The outline coordinate of the femoral implant is set up so that whenever the burr tip coordinate is equal to any of the outline coordinate of the femoral implant. This triggers a warning message and stops the drill motor.

**Figure 6:**
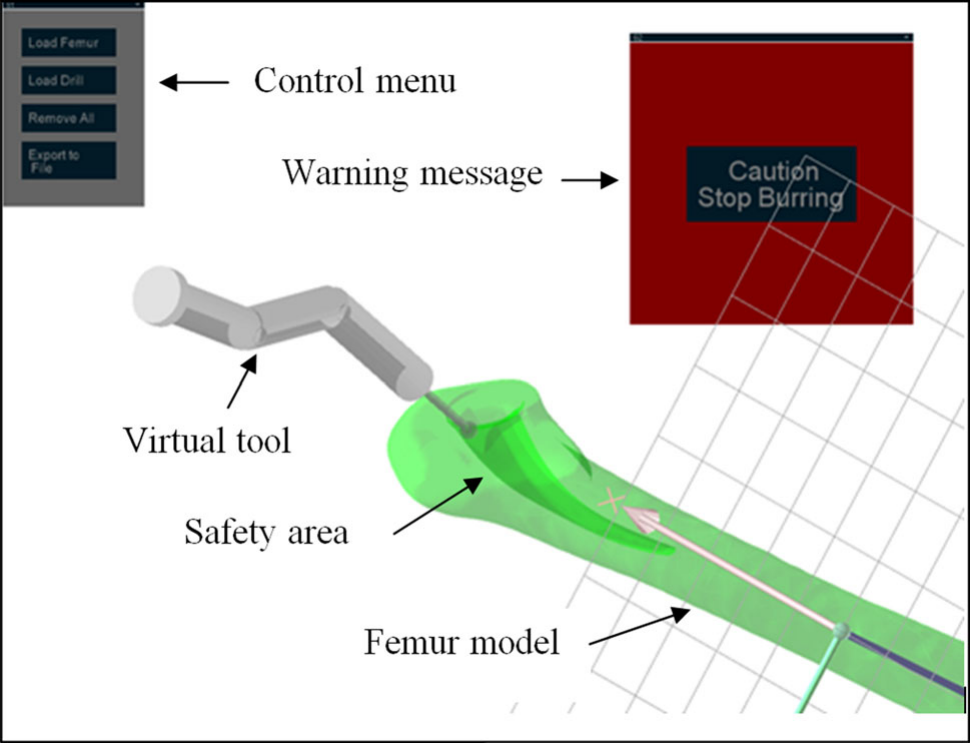
A graphical user interface (GUI) of the navigation system.

This process is followed by a pre-operative planning of computer assisted orthopaedic surgery (CAOS), by means of which a digital image of the bone and surgical tools is obtained, and then mapped onto the navigation system. The CAD model of the drill 3D is the virtual object (VO) in the navigation system. The VO has its coordinates and orientation mapped in the navigation system. The coordinates and orientation data are used to register the VO to the surgical objects (SOs). It is done by synchronizing the coordinates and the orientation of the VO as it follows the coordinates of each SOs. This activation enables a real-time position tracking of the surgical objects virtually on the monitor, as shown in Fig. 6.

A navigation system of the flexible drill sheath is also developed using the same programming language as the mapping system. A graphical user interface (GUI) is developed to guide the user through the navigation steps when milling the femoral canal using the flexible drill sheath.

**Figure 7:**
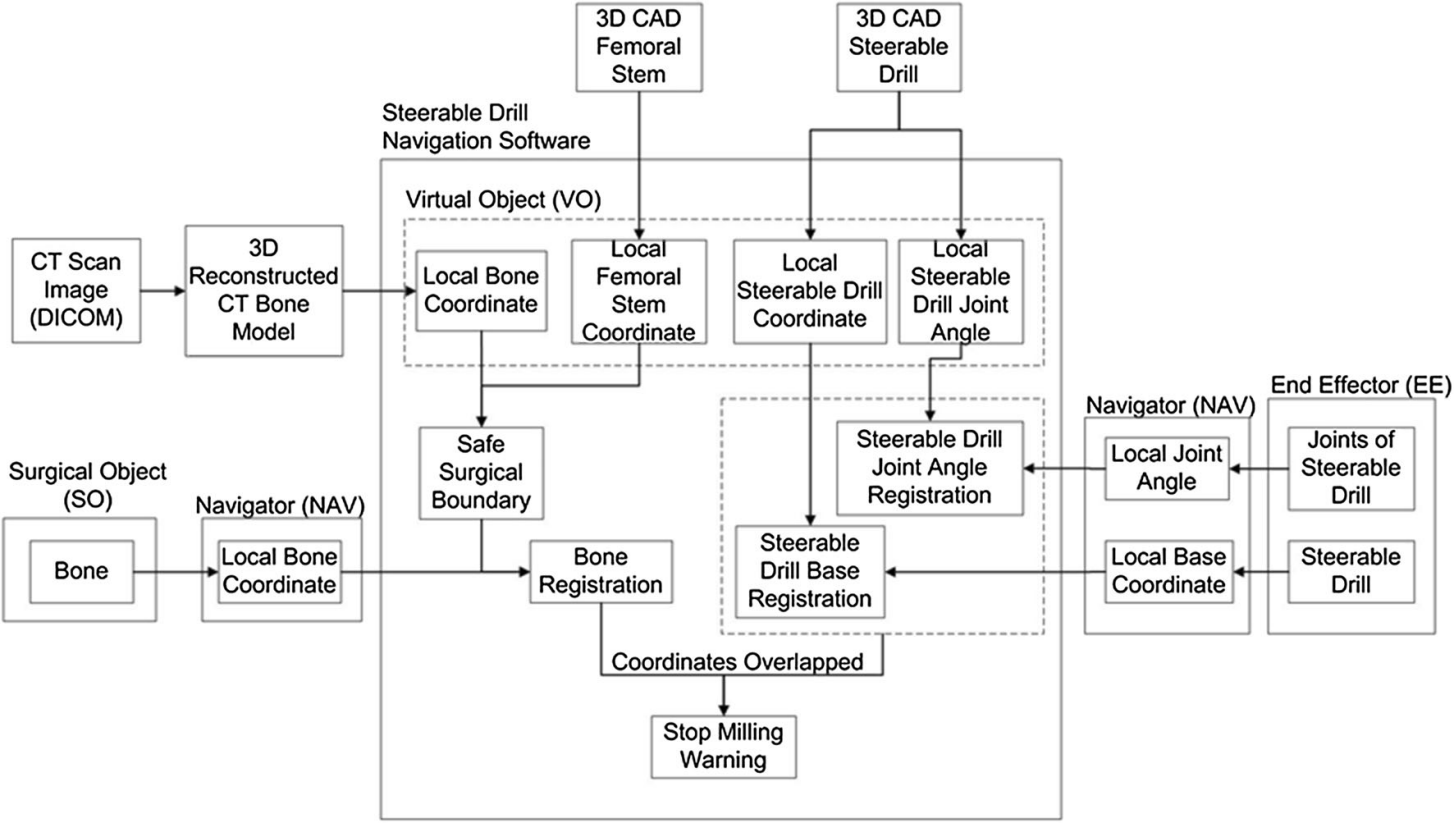
Flowchart of the navigation system in reference to basic concept of CAOS that divides the system into surgical object (SO), virtual object (VO), Navigation (NAV), and end effector (EE).

The concept of this navigation system is illustrated in the flowchart in Fig. 7 in reference to basic concept of CAOS.^21^ The end effector (EE) consists of the base of the drill and the flexible tip by which these two are tracked *via* a hybrid tracking system. The navigator (NAV) consists of a LED tracking camera (optical tracking) and rotary encoders at the flexible drill joints (encoder tracking). The NAV also tracks the surgical object (SO) which is fixed in femur bone. Streaming data from NAV is then registered to VO in the navigation software. The virtual models are reconstructed into 3D models of femoral stem and the drill manipulator. These virtual models have their own local coordinate systems by which are registered with the coordinate systems of SOs and EE. Should these two objects’ coordinates intersect with each other, it will trigger a warning message to stop milling as safety measure to not mill beyond the surgical boundary.

## Experiment

### Experiment Setting

**Figure 8:**
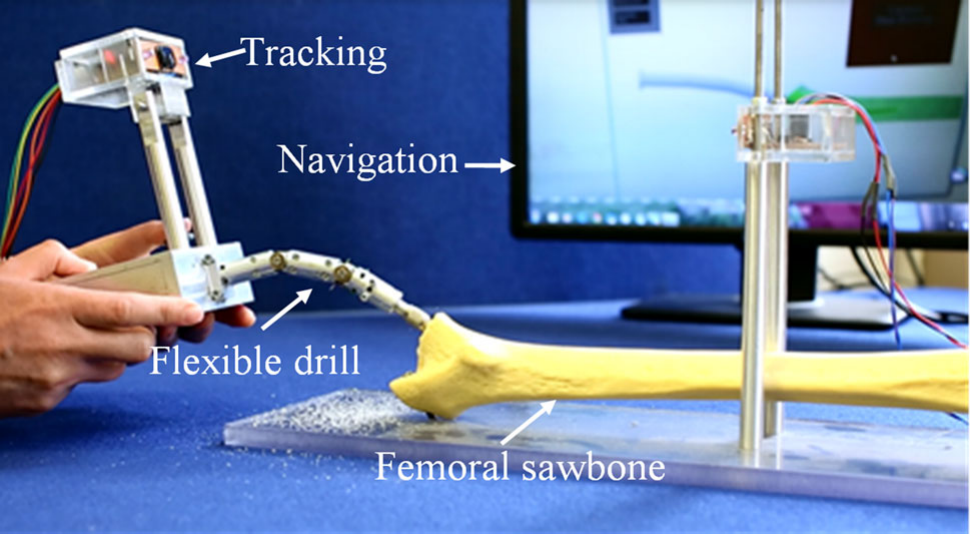
System setting for the flexible drill and its navigation system in a sawbone test rig.

This section presents an integrated flexible drill and its navigation system, shown in Fig. 8, which is tested on sawbone models. There are different levels of integration including mechanical assembly, embedded position sensing and optical tracking, mapping and navigation. In this system, the major part of the mechanical integration is to ensure a reliable mechanical structure of the flexible drill and a robust motor control. The mechanical structure is designed to allow enough space for the rotary encoders to be embedded in the segments and the optical tracking devices to be mounted on the base as a handle. Regarding the software integration, all the virtual models and tracking information are integrated into a unit framework for an easy to use shown in Fig. 6. This paper shows the friendly graphics user interface, which would make the surgical orientation and equipment handling easy for the surgeon. The navigation system provides real time guidance to a surgeon during the procedure of total hip arthroplasty with following function.

- Load the femur 3D model and femoral stem 3D model and get their coordinates
- Set up the safe surgical boundary by planning the femoral stem 3D model in a correct position
- Load the flexible drill model and get its coordinate
- Register the virtual object with surgical objects by integration of optical tracking systems
- Register the joint angle tracking and link it with the position of the drill base
- Start to mill the femoral canal. The drill motor will stop when the burr tip touches the boundary
- Put the femoral stem implant to the milled femur.

In this test rig, the hip sawbone is fixed on a platform at which a tracking unit is placed at its geometric centre, providing the global coordinate information. The motor box of the flexible drill acts as a handle for the surgeon. During the procedure, the thumb stick is used to bend the flexible tip to the proper angle to fit in the curvature tunnelling. An optical tracking unit is mounted on the handle to provide 6 DOFs tracking information which refers to the location of the handle.

The procedure is guided by the navigation system shown on the computer screen. Mapping enables surgeons to view virtually both the 3D model of femur and the 3D model of a femoral stem, thus enabling the surgeon to position the femoral stem inside the 3D bone model precisely. Also, both the femur model and the femoral stem’s coordinates are linked together, providing a virtual interaction between the models.

### Drilling Experiment

The experiment is carried out by using the flexible drill and its navigation system for femoral milling in THA. Initially, the flexible drill underwent usability and functional tests to check whether it can function as intended to drill a curved tunnel. At this stage, the sheath is attached to a conventional drill and the material to be drilled is made by sawbones or some other objects of the same material. The second step involves tests on the sawbones to evaluate the accuracy of the positioning and the orientation of a femoral stem relative to the pre-operative plan and its alignment with the acetabular cup. A standard size sawbone of a left human femur is used as the sample for this test. The bone is fixed at a bone fixture rig by means of screws. At the steps of the test shown in the attached video, the femoral head is cut off. Then, the drill/burr tip is pushed forwards to mill the shape designed, adjusting the orientation and position. To proceed with the test, the burr tip is first placed adjacent to the greater trochanter to check and confirm that the flexible drill has been registered and tracked in. The motor drill is then turned on when the femoral neck is cut from the femoral head. At this step, the femoral canal is created using the flexible drill, following the pre-planned cutting area with visual feedback from the navigation interface. During this procedure, the milling process stops whenever the ‘Stop Milling Warning’ message has been triggered and then resumes after taking out the flexible drill from the femoral canal.

**Figure 9:**
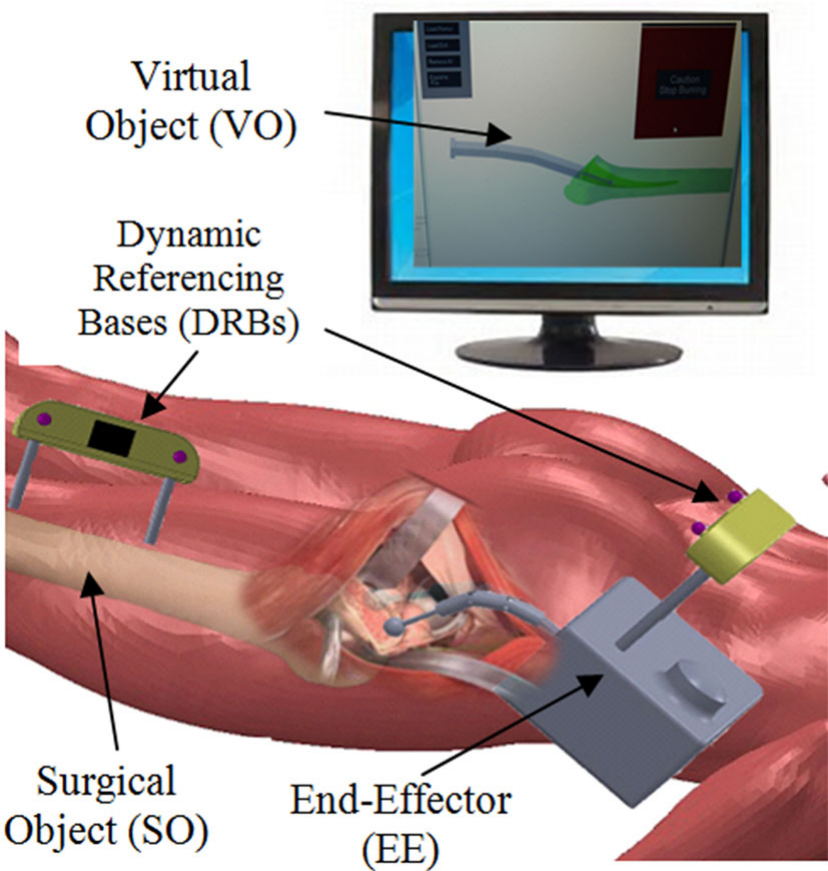
Conceptual clinical setting for THA using the flexible drill and its navigation system.

In real clinical setting proposed in Fig. 9, one tracking unit will be fixed in patient femur as a dynamic referencing base (DRB). The two most common type of registration technique are paired points technique and surface registration technique. Surface registration technique is further divided into two, which are anatomical landmark technique and fiducial-based technique such as using bone pins. Both of these techniques require defining of the anatomical landmark, and image segmentation in the pre-operative planning.^14^

## Results and Discussion

**Figure 10:**
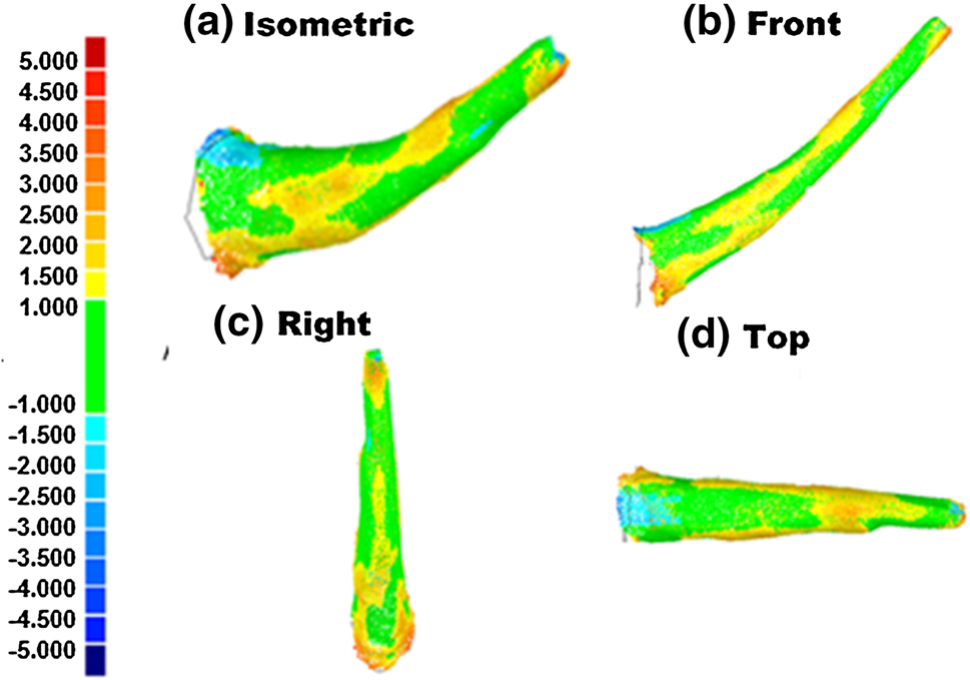
The chromatogram used for an analysis of the milling procedure.

Once the milling test is completed, the sawbone is sent for CT scan imaging, in order to analysis the milling outcomes. The CT image taken is then reconstructed into a 3D digitized geometry by a commercial software package called MIMICS (Materialize NV, Belgium). The 3D digitized geometry is imported into analysis software Geomagic Qualify 12 (Geomagic^®^) to isolate the milled area boundary from the whole geometry. The pre-planned cut area is also imported into Geomagic Qualify 12 (Geomagic^®^) to act as a reference template, while the milled area boundary is acted as a test object. The best fit alignment method and an iterative closest point algorithm is used to best fit the objects. The chromatogram is generated automatically, as shown in Fig. 10. The chromatogram represents the deviation of the test object from the reference template, in which a deeper colour means a larger deviation. In the figure, the range is set as ± 5. 0 mm. Deep red represents + 5.0 mm and blue represents − 5.0 mm. The analysis covers the maximum positive and negative deviations and the standard deviation.

Figure 10 shows the chromatogram of a femoral sample’s cut area in four views at front, top, right, and isometric. The colour ranges from green that indicates less than 1 mm of deviation to red and blue in colour, which indicates more than 5 mm of deviation overcut and undercut respectively. The right view in Fig. 10 shows that it has two protrusions at the front surface of the cut area due to presence of overcut. It can be clearly seen that the peak of each protrusion was red in colour. Besides that, the middle section of superior surface overcut tapered and extended towards the tip of the cut area. The front view in Fig. 10 shows a mixture of cut of less than 1 mm (green colour), and overcut of between 1  and 1.5 mm (yellow colour). The top view in Fig. 10 shows that it has overcut at the middle section of superior surface that extended to the front surfaces. These similarities signify the repetition of overcut and/or undercut at certain area in relative to the pre-planned 3D model.

**Figure 11:**
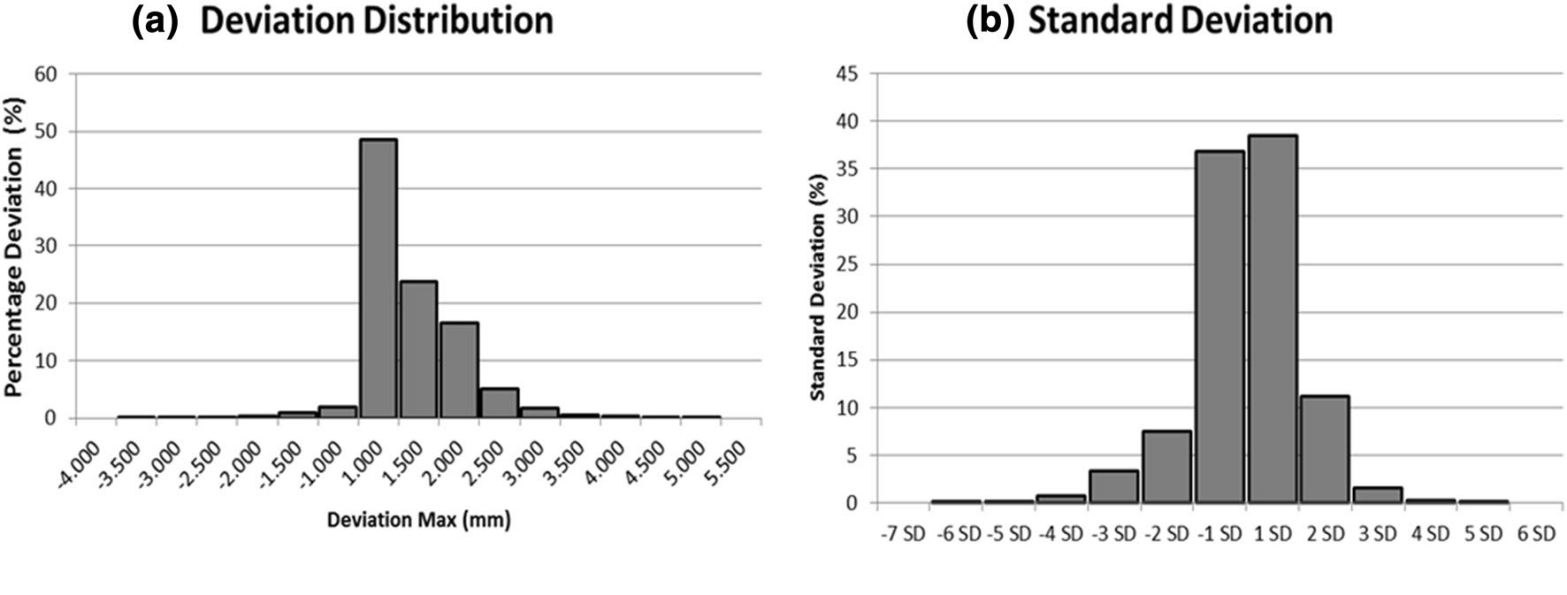
(**a**) Percentage deviation distribution of point cloud data of cut area of the femur sawbone; (**b**) Standard deviation of point cloud data of cut area of the femur sawbone.

The accuracy of the milled area boundary is evaluated using Geomagic Qualify software to establish a 3D deviation profile for the test object against a reference template. It has been found in Fig. 11(b) that 75.232% of the point cloud data of the milled area boundary is within ± 1 SD (0.864 mm) of the pre-planned cut area; 93.924% of the point cloud data is within ± 2 SD (1.728 mm). This indicates that the majority of the cloud data from the geometric shape of the milling boundary is within 1.728 mm (± 2 SD) in relation to the pre-planned cut area. Hence, the accuracy of the navigation system is within 1.728 mm. However, there is still 1.813% of the point cloud data exceeding the positive deviation value, and 4.264% exceeding the negative deviation value. The geometric variations of the cut area in comparison with the outline of the femoral stem from a pre-plan of navigation software are measured and presented using a deviation analysis. The analysis of the deviation confirmed that the flexible drill system is able to mill inside a femoral bone with a deviation between the cut areas and the outline of the femoral stems from navigation software that is in the range of − 0.759–1.151 mm and is slightly off from the acceptable clinical range of 1 mm.

The detailed deviation analysis quantifies the deviation of the cut area to the outline of the femoral stem from the navigation software. It is found that a small portion of the deviation is more than 2 mm (7.477 ± 2.857% deviation between 2 and 3 mm, 1.050 ± 0.317% deviation between 3 and 4 mm, and 0.156 ± 0.237% deviation above 4 mm). This means that the cut area slightly deviated by up to 2 mm from the outline of the femoral stem calculated by the navigation software.

As seen in Fig. 11, the most significant proportion of these larger discrepancies came from the distal end of the cut area and the proximal part of the cut area. The main reason of the errors is that the femoral stem used is the tapered femoral stem that has a diameter of less than 6 mm towards the end of the tapered tip. But the mill bit used has a diameter of 6 mm, therefore, the smallest cut in terms of the diameter can only be 6 mm. This resulted in the distal tip of the cut area having a greater error due to the fact that mill bit cannot mill the bone smaller than a 6 mm diameter. These errors are similar to ‘imperfect drilling characteristics’^7^ in robotic-assisted skull base surgery. However, robot kinematic error, and mill bit deflection due to the surgeon’s applied force as shown in study that involves a flexible tool, could also be factors contributing to the errors mentioned above.^15^ Apart from that, these errors can be further reduced by using a rotary encoder system that is better than a potentiometer such as fibber bragg grating sensors or a high definition optical rotary encoder system.^19^ Dimensional error due to deflection can be reduced by improving the flexible drill sheath stiffness and utilizing an active compensation method such as estimation of a mill bit deflection by measuring the force applied by surgeon. The large discrepancies at the proximal part of the cut area is caused by chipping of the sawbones due to the difference in hardness between the sawbones’s outer layer’s resin and the inner resin compound. The outer layer’s resin is harder and had a minimal hollowed structure, while the inner resin is softer with more a hollowed structure. The root mean square (RMS) obtained showed an indirect correlation with the magnitude of deviations and it signifies the accuracy of the system in milling the cut area. However, since it has an indirect correlation with the magnitude of deviations, high deviation regions on red and blue colour at the distal end of the cut area contributed to the larger value of RMS, hence reducing the accuracy of the system. Finally, a possible reason for reducing the accuracy of the system is its setting of their DRBs. In the sawbones experiment, one tracking unit is fixed in the femur as a DRB. As the SO in real clinical setting could be moving, such non-fixed SO would affect the positioning accuracy. Thus, an additional fixed tracking unit is introduced to be a globe reference for the system to track both DRBs attached in the patient’s femur and the surgical drill.

The femoral stem implant is a solid body which will fit into a cavity. It will sit where the area of the cavity comes in contact first and that will be the majority of the contact area. Although the accuracy of the navigation system is 1.728 mm, the difference is small since the defect is on the inner side of the aim (− 1 SD) as shown in Fig.11(b). This error results in a cavity which is 1 mm less than the implant dimensions, thus reducing the chance of burring the cortex too much, and the implant will have a nice press fit stability if the implant is an uncemented implant.^18^ If the femoral stem implant is a cemented implant, the accuracy of 1.728 mm is good since there is space of nearly 2 mm for filling the cement and press to fit the implant into the desired position.

## Conclusion

In this paper, a novel flexible drill system for orthopaedic surgery has been presented and demonstrated. In THA, CAOS is currently practised only with acetabular cup positioning, while, femoral stem positioning still uses the hand-rasping method instead of femoral milling. Although some devices have used flexible drills, they are not robotic systems and only can be tracked by using X-ray images. Our new system has demonstrated its ability to perform femoral milling using a unique flexible and steerable drill coupled with a novel tracking and navigation system. As the flexible drill tip is not trackable *via* an optical tracking system inside the bone, this paper has presented a novel hybrid tracking and multimodality navigation system for guiding the flexible drill tip.

Experiments in sawbones have proved that the new system can not only provide a new capability but also the accuracy of the milling reaches a satisfactory level in femoral milling. The applications of this robotic system are not only for the purpose of carrying out femoral milling in MIS THA, but also for other skeleton related procedures such as tunnel drilling in ACL reconstruction, milling in revision of arthroplasty, and drilling in head and neck surgery. Further research is needed in order to put this concept into practice in a clinical setting.

## Electronic Supplementary Material

Below is the link to the Electronic Supplementary Material.

Supplementary Material 1 (Wmv 109539 kb)
